# Data Archive for the BRAIN Initiative (DABI)

**DOI:** 10.1038/s41597-023-01972-z

**Published:** 2023-02-09

**Authors:** Dominique Duncan, Rachael Garner, Sarah Brinkerhoff, Harrison C. Walker, Nader Pouratian, Arthur W. Toga

**Affiliations:** 1 Laboratory of Neuro Imaging, USC Stevens Neuroimaging and Informatics Institute, Keck School of Medicine of USC, University of Southern California, Los Angeles, CA USA; 2 Department of Neurology, University of Alabama at Birmingham, Birmingham, AL USA; 3 Department of Neurological Surgery, UT Southwestern Medical Center, Dallas, TX USA

**Keywords:** Databases, Computational platforms and environments, Data integration

## Abstract

Data sharing is becoming ubiquitous and can be advantageous for most biomedical research. However, some data are inherently more amenable to sharing than others. For example, human intracranial neurophysiology recordings and associated multimodal data have unique features that warrant special considerations. The associated data are heterogeneous, difficult to compare, highly specific, and collected from small cohorts with treatment resistant conditions, posing additional complications when attempting to perform generalizable analyses across projects. We present the Data Archive for the BRAIN Initiative (DABI) and describe features of the platform that are designed to overcome these and other challenges. DABI is a data repository and portal for BRAIN Initiative projects that collect human and animal intracranial recordings, and it allows users to search, visualize, and analyze multimodal data from these projects. The data providers maintain full control of data sharing privileges and can organize and manage their data with a user-friendly and intuitive interface. We discuss data privacy and security concerns, example analyses from two DABI datasets, and future goals for DABI.

## Introduction

Human intracranial recordings are used to study a variety of neurological disorders, such as epilepsy, stroke, neuropsychiatric disorders, and Parkinson’s disease and other movement disorders. The Data Archive for the BRAIN Initiative (DABI) provides a platform of networked and centralized web-accessible data archives to capture, store, curate, and share data related to the Brain Research Through Advancing Innovative Neurotechnologies® (BRAIN) Initiative^1^ proposals that collect human intracranial neurophysiological data for the broader scientific community (https://dabi.loni.usc.edu). DABI was created at the Laboratory of Neuro Imaging (LONI) at the University of Southern California by Drs. Arthur Toga, Dominique Duncan, and Nader Pouratian and is funded through the National Institutes of Health as part of the BRAIN Initiative.

Data sharing can be a valuable force to accelerate scientific discovery. Intracranial neurophysiology studies are often exclusive in their design and methodology, which presents unique challenges for data sharing. Given the invasive nature of these studies and the involvement of potentially vulnerable neurosurgical patients, costs and recruitment for such studies are uniquely challenging, as well. Small patient cohorts reduce the statistical power necessary to validate the safety and efficacy of invasive devices and to identify candidate patients, target brain regions, and recording/stimulation parameters. Given the relative rarity of these recordings compared to recordings in model systems, there is an even greater imperative and need to share across research institutions. For these reasons, interest surrounding data sharing to expand patient cohorts has grown in recent years.

**Fig. 1:**
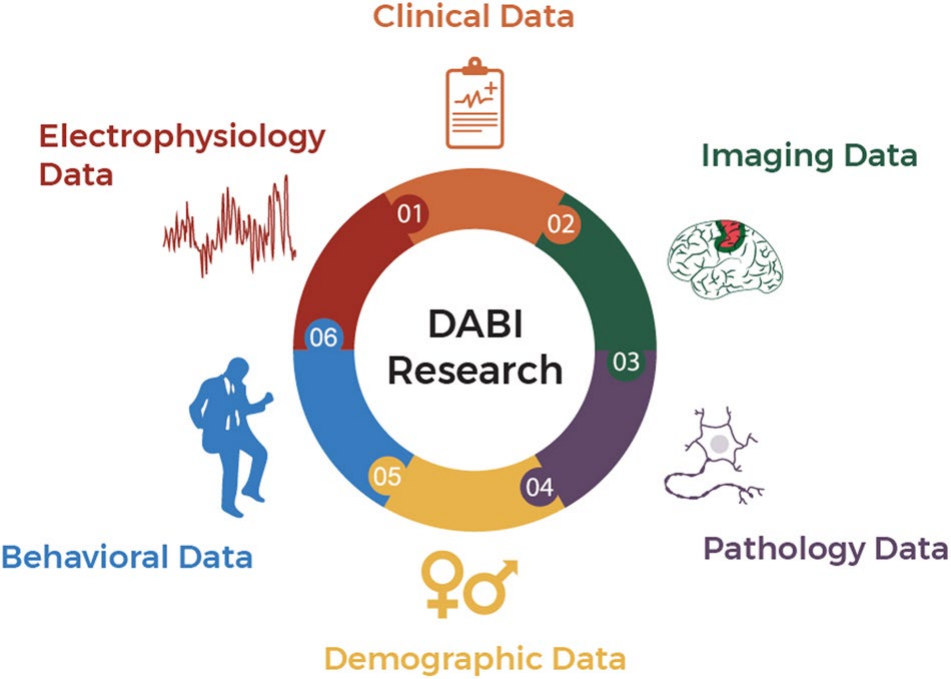
An overview of DABI data types. DABI allows data partners to upload electrophysiology, clinical, imaging, pathology, demographic, and behavioral data.

There are clear advantages to facilitating the sharing of these valuable data collections, as evidenced by the significant successes of multi-site data sharing related to Alzheimer’s disease^2,3^ and Parkinson’s disease^4,5^, among other neuroscience efforts. Expanding available cohorts for multi-site analysis also expands the likelihood of identifying generalizable findings for exploratory investigations, as examining cognitive function within only one pathology or a narrow clinical phenotype may yield clinical confounds. DABI fills this scientific need by providing a centralized database for human intracranial neurophysiology recordings and related data – clinical, imaging, pathology, demographics, behavioral, and scalp electrophysiology (Fig. 1) as well as a wide variety of associated data formats. DABI ingests, harmonizes, aggregates, stores, visualizes, and disseminates a wide variety of data types. And importantly, provides for granular search as part of its interface.

While standardizing the format for organizing human intracranial neurophysiological data has gained some traction with the Brain Imaging Data Structure (iEEG-BIDS)^6^, previous efforts to create centralized data archives for human intracranial neurophysiology data have not been widely adopted due to many challenges, such as large file sizes, persistent varying formats, privacy constraints, and funding.

The European Union-funded EPILEPSIAE database was made publicly available in 2012 and provides long-term scalp and intracranial electroencephalography (iEEG) recordings with annotation and metadata of 275 patients^7^. Access is restricted to scientific groups that financially contribute to the maintenance of the database. The National Institute of Neurological Disorders and Stroke-funded cloud-based platform, IEEG.org, contains over 1200 human and animal (dog, mouse, rat, sheep, and primate) datasets that includes neuroimaging, EEG, electrocardiogram, and clinical data^8^. This platform uses Amazon cloud services for data storage. However, one of the major challenges is the need to secure enough resources, long term, to sustain the effort beyond the initial funding period so that the platform can truly bring value to the broader scientific research community^9^. Moreover, coupling such data archives with analytic tools can help researchers with their analyses and increase reproducibility. Existing data archives such as IEEG.org support code dissemination by linking users to public GIT repositories or hosting downloadable files within a stored dataset. However, this requires users to have code and data on local machines and have access to sufficient computational resources to perform the required analyses.

In developing DABI, we have taken these concerns into consideration and applied our experience in other types of data sharing platforms^10,11^. We have built DABI, using our existing tools and unique resources, with the goal of maintaining the archive after funding ends without charging user fees for data access. LONI has dedicated itself to maintaining data archives and hosting software even after funding windows close: all former LONI projects from the last 30 + years have been maintained continuously. With this mindset, we hope to ensure the perpetuity of the archive so that it remains beneficial to the scientific community.

## Methods: DABI Architecture

**Fig. 2:**
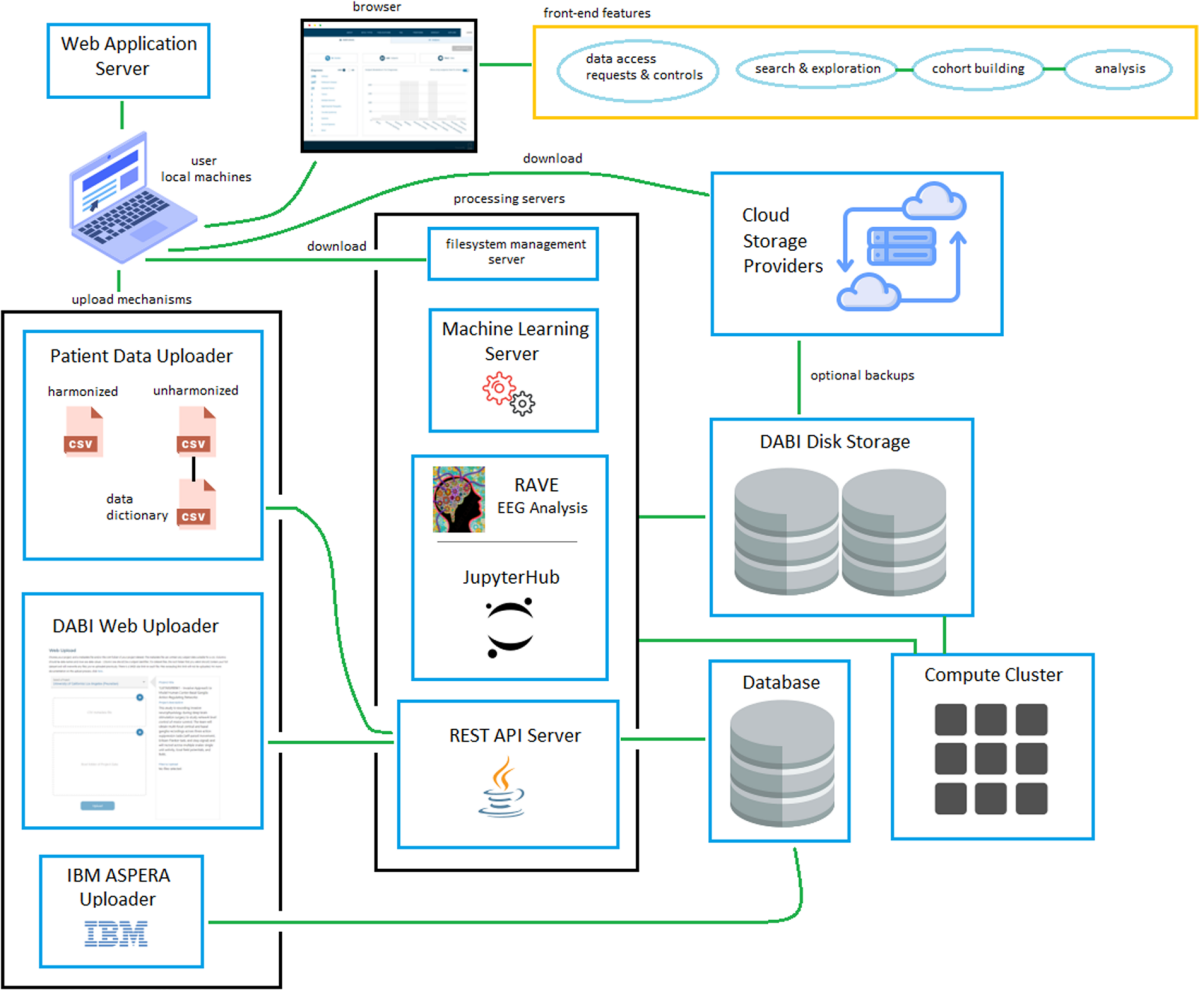
A schematic of DABI’s system architecture. Users access the DABI web application via their local machine. The user interface includes features to enable users to manage data access requests and controls, explore available datasets via search, build relevant cohorts, and then perform analysis on saved cohorts. Several upload methods are available. The DABI Web Uploader is recommended to upload comma-separated value (CSV) metadata and smaller datasets that are likely not subject to disruption due to internet time-outs. Larger datasets can be uploaded via ASPERA, the latest Health Insurance Portability and Accountability Act (HIPAA)-compliant software utility from IBM. A forthcoming feature, the Patient Data Uploader, will allow users to map unharmonized metadata into a standard ontology, or upload unmapped data that other users can map as part of cohort building. Users may also connect existing cloud storage accounts with DABI should they prefer cloud-based storage. DABI links with several analytics packages including R Analysis and Visualization of iEEG (RAVE)^26^, an open-source software for iEEG analysis funded by the BRAIN Initiative and Jupyter Notebook^25^, a web-based interface that supports sharable coding and visualization.

DABI is designed to address the full lifecycle of scientific inquiry for BRAIN partners, from secure data ingestion and storage through visualization, analytics, and dissemination. Specific functionalities, seen as an overview in Fig. 2, include (1) data de-identification, protocol detection, and data deposition, (2) data quality assessment, processing of quality assessment results to tag data, and analysis results data integration, (3) mapping of data attributes into a common schema for use in search and visualization interfaces, (4) interfaces to search, select, and download data, (5) integrated processing so that database and compute resources are coupled, (6) a visualization interface for inspecting and comparing data, and (7) a comprehensive website containing training materials, study-related information (e.g., protocols), announcements, and a knowledgebase wherein investigators post questions and receive answers from DABI and the community.

### Data storage

DABI accommodates 2 different models for data archiving. Contributing investigators who prefer to deliver their data to a centralized database can transfer their data for secure storage at LONI (see section 2.2). Under centralized systems, researchers collect cohort data and store the data in a single remote system that allows for convenient storage and retrieval as well as user access controls for sharing data with other specified scientists. This allows researchers to utilize computational tools that they may not have sufficient resources to develop otherwise. The centralized mechanism can be chosen by using Aspera or the Web Uploader to upload data that is stored on DABI servers.

The second model accommodates those investigators who prefer to use cloud-based storage for their data. The cloud storage mechanism can be chosen on the Provider Controls page by linking a cloud storage account. The cloud storage option is achieved by implementing cloud specific linking protocols so that users can connect supported cloud storage service accounts with DABI. Once the cloud account is linked, cloud downloads are achieved by mediating data transfer between the storage provider and the user’s browser. The fees associated with cloud storage are arranged between the data owners and the cloud storage provider. If an institution chooses to end their cloud service or wish to convert to centralized storage for another reason, they can take a cloud snapshot, where a copy of the dataset is stored centrally on the DABI servers.

These two options are available for data providers to choose, and we are agnostic to where each project’s data are stored. Regardless of storage mechanism, data are accessed on the Request Data page and on the DOI (main page) for each project.

### Data upload

**Fig. 3:**
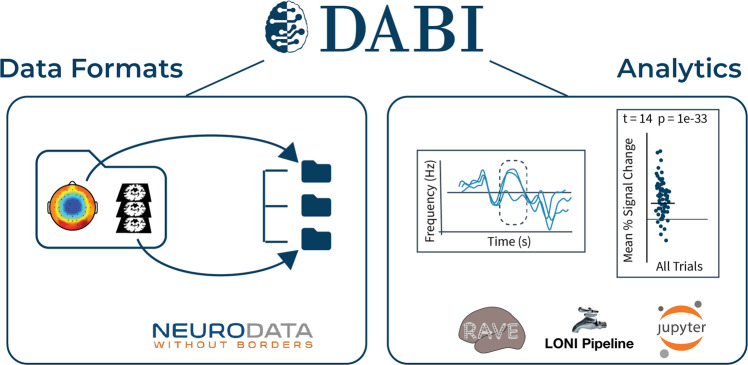
DABI’s suggested data formats and affiliated analytics packages. DABI encourages the use of data formats and specifications consistent with other BRAIN Initiative efforts, including Brain Imaging Data Structure (iEEG-BIDS)^6^, a community-driven specification for imaging and intracranial electroencephalography, which has a tree organization and specific rules for naming, and Neurodata Without Borders (NWB)^12^, a standard format for neurophysiology data and related metadata. Additionally, DABI links with several analytics packages including R Analysis and Visualization of iEEG (RAVE)^26^, an open-source software for iEEG analysis funded by the BRAIN Initiative; LONI Pipeline^22,23^, a distributed system for designing, executing, monitoring, and sharing scientific workflows on grid computing architectures; and Jupyter Notebook^25^, a web-based interface that supports sharable coding and visualization.

Data providers who choose to use the centralized data storage model have 3 options to upload data to DABI. The Web Uploader allows users to upload comma-separated value (CSV) metadata, a blank version with standardized metadata fields is provided on the Web Uploader page for reference, and datasets that are stored according to iEEG-BIDS^6^, a community-driven specification funded by the BRAIN Initiative which has a tree organization and specific rules for naming. This method does not require any additional software to be installed on the providers’ local machines. The uploader validates the BIDS-compliance and anonymizes data to be sure there is no patient history information. Both zipped and unzipped files are accepted. When providers are ready to add new files to their existing DABI data they can simply upload the root folder and only new files will upload without uploading existing data again. DABI encourages use of iEEG-BIDS to ensure comprehensive documentation and streamlined ingestion to standard data processing pipelines. DABI also supports file formats stored in the Neurodata Without Borders (NWB) data format, a BRAIN Initiative funded project to standardize neurophysiology data and related metadata^12^. These data formats and the analytics package linkages that we use are shown in Fig. 3.

Datasets that are not stored in iEEG-BIDS can also be uploaded using ASPERA, the latest Health Insurance Portability and Accountability Act (HIPAA)-compliant software utility from IBM. ASPERA is an extremely fast—10–100x faster than traditional file transfer protocol (FTP)—and lightweight file transfer client and is not subject to limitations of web browsers. This upload method requires no cost installation of the client ASPERA on a local machine, followed by a request for connection and host credentials from DABI, which include file paths and storage locations on the DABI servers. Once installed, providers simply log in and select files to transfer. No file structure or naming requirements are involved and data deletion from DABI can occur at any time. Data providers may also use their own secure shell (SSH) or secure FTP (SFTP) File Transfer Client. DABI will provide the necessary credentials for data providers to access their project’s home directories on the DABI server. This method does not have file structure or naming requirements, and providers the most flexibility for transferring data.

### Security, data de-identification, access control, and data backup

Access control and encryption are provided to support a wide variety of data sharing policies. Each data provider that contributes data to DABI has consented their patients through permission of their Institutional Review Board, and data owners have the responsibility to remove protected health information (PHI) prior to data upload, though we have some built-in precautions to ensure that HIPAA regulations are followed using our integrated data de-identification components. Uploaded data are immediately available for viewing and download by approved users (if private) or the public, so it is critical that data are de-identified prior to upload. Specific guidelines to reduce the chance of PHI transmission include (1) video and audio uploads are not accepted formats at this time, (2) de-facing procedures are encouraged to reduce patient recognizability and combat automated reverse facial detection algorithms, and (3) use of external de-identification software to remove PHI from standard imaging formats such as NIFTI and DICOM. There is also a built-in tool through the Web Uploader that does allow users to de-identify MRI during upload as an optional step. If this is selected, users can review the changed/removed fields during upload. Imaging data are de-identified to exclude header fields that may contain PHI, including patient name, study date, referring physician, and institution name, among other fields. Any dates are also revised to include only month and year, excluding specific days of birth, intervention, surgery, etc. There is an efficient mechanism for tracking the status of all datasets and providing an audit trail so that investigators know who processed the data when and how. In addition, this audit trail helps to guarantee that all researchers can easily be acknowledged for their contributions. Detailed sharing capabilities are defined by each site with designation of which components are shared at which level. Sharing levels will include (1) site specific, (2) project specific, and (3) public.

We ensure an encrypted transfer to DABI servers using https and Aspera. We provide immediate and uninterrupted access to data as dictated by data use agreements from participating investigators. Furthermore, we have developed functionality to test the speed and reliability of investigators’ network connections and provide recommended download methods based on the test results.

### Data harmonization

To make DABI data sharing most useful across different projects and sites, common data elements, data dictionaries consistent with other BRAIN Initiative efforts, and aliases have been adopted wherever possible. The Neuroimaging Data Model (NIDM)^13,14^ aims to improve metadata precision, especially in the context of experimental design, data acquisition, and analytic workflows. NIDM has worked towards a standard ontology, hosted by SciCrunch with support by NeuroLex, with over 400,000 terms hosted^15–19^. In association, OpenNeuro, a repository for hosting and sharing BIDS imaging datasets, has adopted language to define high-level concepts to annotate datasets^20^.

Currently, we do also accept non-BIDS datasets to support institutions at which standard specification adoption is still ongoing. If data are not BIDS-compliant, we do not convert or modify the original datasets to adhere to BIDS, as conversion from potentially proprietary or custom data formats requires a highly detailed knowledge of the study and data acquisition. However, we do ask that users provide one additional file (that is not part of the uploaded dataset) that includes harmonized metadata fields to facilitate searchability of data on the Explore Page. These variables were proposed by data partners, are at the subject or project level, and allow users to search for datasets that meet specific criteria of interest. A template CSV with potential variables is available for download on the Web Uploader page. Variables include subject ID, gender, age, diagnosis, handedness, interventions, region of interest/electrode location, device(s) model, etc. These CSVs are then internally converted to JavaScript Object Notation (JSON), a highly adaptable data-interchange format that stores text as attribute-value pairs and arrays^21^, which is also the candidate file type due to its utilization by iEEG-BIDS^6^. We have built this together with the data providers to accommodate laboratory needs, and we continue to work closely with them to adapt these features.

### Data access management

Investigators establish their own data use requirements and policies, which may vary for users at different laboratories and projects. We have created a flexible system with various levels of granularity so that access control rules and consequent authentication systems match the needs of the different projects. Data providers often choose to share data after a publication, so we have given data providers the ability to do this and to choose a date for making those data public if they know the date in advance.

DABI has also integrated automated digital object identifier (DOI) generation. Anytime a project or subproject (a subset of files from a project) is created, DABI automatically registers a unique link with DOI.org. This link grants view access to the dataset homepage, which includes a customizable dataset description and visualization of the dataset’s file tree. However, users must be logged in and have explicit data access permission (granted by the PI) to allow data download. Data providers may also be given a publicly available link to their dataset that does not require an account for access; this link includes an embedded token that allows the accessor to download data without logging in. This can be useful to include for anonymized peer review or publications.

Most importantly, the data providers always remain in full control of data access.

### Querying data and cohort creation through the explore page

**Fig. 4:**
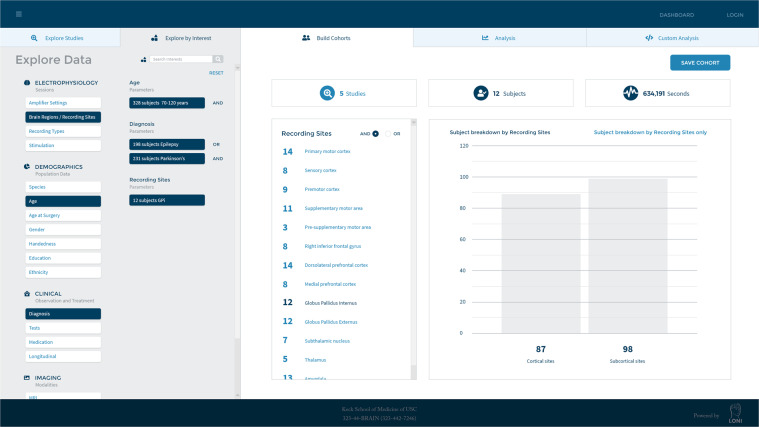
A screenshot of the DABI Explore Page. The Explore Page is a web-based user interface that allows users to query project metadata. Users can search amongst common variables such as age, diagnosis, and recording sites.

Although all data remain private until explicitly made public by a PI or delegate, all metadata are searchable through the Explore Page. We have linked this integrated graphically controlled processing system so that the results of queries can be explored further prior to data download. Patterns and trends that may be observable across projects can be visualized and plotted using tools that we provide that are coupled with the data portal. We have developed techniques and standards to import and interlink data and metadata from a variety of different modalities, support highly flexible search and browsing of these data (Fig. 4), and enable linking analyzed results to raw data along with their provenance.

Investigators can query data based off specific filters, including gender, diagnosis, age, recording location, and data modalities available. These filters can then be used to create cohorts that can be used in downstream analyses.

### Integrated analytic tools

By centralizing an enduring data archive, we allow the broader neuroscience research community to access and thereby analyze the data from various BRAIN Initiative projects. All information pertaining to data acquisition, quality control, pre-processing, and analyses are captured and retained, providing a comprehensive history and provenance to the data. Data provenance includes timestamped raw data with timeline noting data upload revisions and versions, preprocessed data (provided by data collectors or produced by users within associated analytic tools), saved cohorts, and analysis workflows saved by users. We have pioneered innovative standardization/co-registration references, fully supported by novel image and electrophysiology processing methods, to extract candidate biomarkers from the diverse data to address the specific projects’ goals. Spatial descriptions and co-registrations of regions of interest are made according to detailed coordinate/imaging maps of the brain, co-registered to sensors, such as implanted or scalp electrodes, when possible. With the aid of the LONI Pipeline^22,23^ that is integrated into DABI, much of this work is automated. Not only is a well-curated and standardized multi-modal data set facilitating the development of models of various diseases, but it is also ensuring that such models are statistically significant and validated.

Data trends and correlations can then be calculated in DABI, without downloading raw data. Integrated software and analytics include image visualization, quality control^24^, LONI Pipeline^22,23^, Jupyter^25^, R Analysis and Visualization of intracranial EEG Data (RAVE)^26^, and a variety of statistical tests. RAVE allows users to visualize intracranial EEG (iEEG) recordings and apply various dimensionality reduction and statistical methods to analyze these large iEEG datasets^27,28^. Investigators maintain complete ownership and control of their data. Unaffiliated users must be granted access from PIs to download raw data or conduct analysis using DABI’s built-in analytics.

## Results: Sample Analyses within DABI

### Performing statistical analyses

DABI has integrated a statistical workflow to allow investigators to perform exploratory analyses without needing to download any data to their local machine. The pipeline supports both nominal (e.g. diagnosis) and numeric variables (e.g. spectral analysis). The analysis framework eases identification of correlations between multiple variables and a target parameter via batch processing. Depending on the data distributions, and whether the independent and dependent variables are numeric or nominal, appropriate statistical tests are applied. For example, to evaluate the relationship between movement disorder diagnoses and band power, Welch’s T-test would be performed between the diagnosis and average band power for each frequency range. Comprehensive documentation of the statistical tests available within DABI can be accessed via the DABI site^29^.

**Fig. 5:**
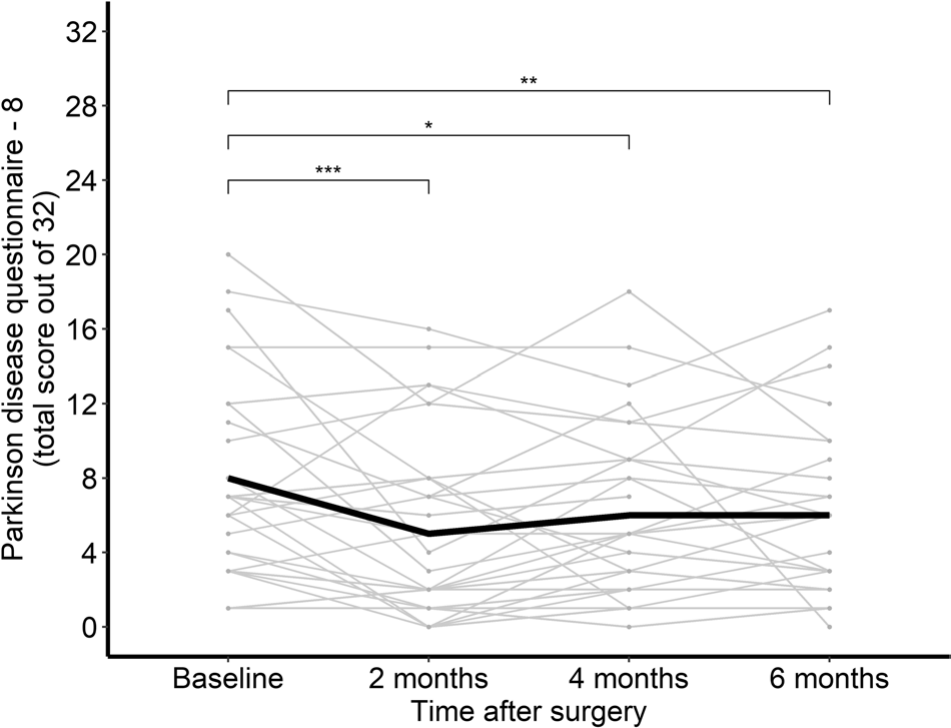
Sample statistical analysis performed within DABI. Linear mixed effects regression assessing Parkinson’s Disease Questionnaire-8 (PDQ8) score across time for 31 patients who underwent unilateral directional subthalamic nucleus deep brain stimulation for Parkinson’s disease. Data provided by Dr. Harrison Walker at the University of Alabama at Birmingham. Health related quality of life scores from PDQ8 decrease with treatment at multiple time points versus preoperative baseline (p-value: 0.005). ***p < 0.001, **p < 0.01, *p < 0.05.

In one analysis we utilize data collected by Dr. Harrison Walker at the University of Alabama at Birmingham^30^. His BRAIN Initiative study investigates directional lead technology with the goal to determine electrophysiology biomarkers that best predict the optimal combination of active contacts with directional DBS electrode technology. This dataset for 31 patients includes intraoperative electrophysiology, imaging, and longitudinal motor and neuropsychological testing. Within the DABI analysis workflow, users can explore various methods to assess symptom improvement over the course of the study. In this example, we perform a linear mixed effects regression to model the patients’ Parkinson’s Disease Questionnaire-8 (PDQ8) score across the session timeline (e.g. preoperative baseline, 2 month follow-up, 4 month follow-up, and 6 month follow-up). This analysis (Fig. 5) illustrates that PDQ8 scores decrease under current treatment (p-value: 0.005).

### Machine learning ecosystem within DABI

DABI also supports advanced supervised data exploration through its innovative machine learning (ML) ecosystem. The ML framework includes supervised learning to perform classification and regression with standard algorithms in the H2O AutoML library^31^, an open-source, R and Python-supported infrastructure for scalable ML. Supported models include General Linear Model, Random Forest, XGBOOST, Gradient Boosting, Deep Learning, and Stacked Ensemble. DABI’s automated ML ecosystem has been designed in a way such that users can build models within minutes even without knowledge of programming by inputting specific parameters, such as the number of folds for k-fold cross-validation or whether to perform oversampling to balance an unbalanced dataset. Analysis results such as variable importance and area under the curve measures are automatically visualized to allow users to evaluate model performance.

**Fig. 6:**
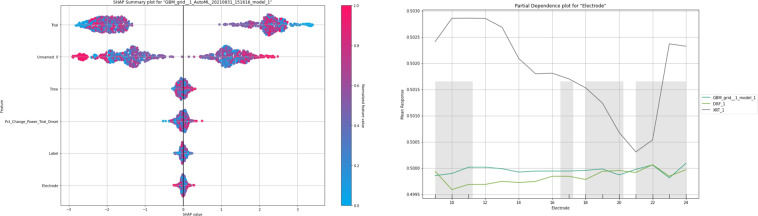
Sample explainability figures generated by DABI’s Auto Machine Learning (Auto ML) pipeline^31^. In this analysis we used multiple algorithms such as Random Forest and Gradient Boosting Machine. Left shows a Shapley Additive Explanations (SHAP) plot, which quantifies the contribution of each feature on overall classification or prediction, in this case the task type. In this sample analysis, time, percent change of power trial onset, and harmonized electrode location did not have significant impact on performance, but the trial type indicated higher SHAP scores. Right shows a partial dependence plot indicating the mean response performance related to the harmonized electrode number for 3 models: Gradient Boosting Machine, Distributed Random Forest, and Extremely Randomized Trees.

The ML ecosystem is built to allow custom electrophysiology analyses conducted in RAVE to be easily and automatically introduced as variables in ML. In this example, we performed task-based iEEG analyses in RAVE for 26 Parkinson’s disease and Dystonia patients from Dr. Nader Pouratian’s study conducted at University of California, Los Angeles and UT Southwestern^32^. In this study, Dr. Pouratian records invasive neurophysiology during deep brain stimulation surgery to study network level control of motor control. The team obtains multi-focal cortical and basal ganglia recordings across three action suppression tasks (self-paced movement, Eriksen Flanker task, and stop signal). Electrophysiology data were first preprocessed to remove false trials and perform notch filtering and wavelet decomposition. Then, RAVE power signal outputs and harmonized brain recording regions were introduced into several ML models (Random Forest, Gradient Boosting Machine) to explore whether there were correlations between these variables and the task type (Fig. 6).

### Future goals

By working closely with BRAIN Initiative data providers, we aim to adapt DABI and develop more features that would be beneficial for the data providers. User feedback is critical to this process, and we hope that close involvement by BRAIN Initiative partners will help us iteratively design a useful site for data storage, exploration, and analysis. As more projects are onboarded and more data are shared, we expect the platform will be even more useful for both data providers and other researchers who request access to data. As reach has grown, we have added a Frequently Asked Questions page to the site to support new users. We have also added a feature that allows users to submit questions and requests for features on the contact page to encourage user feedback. We plan to continue to request feedback from users regularly so that we can adapt the platform to their specific needs.

## Discussion

DABI is a unique and innovative data repository designed for the needs of the BRAIN Initiative projects that collect human invasive recordings. It is a platform of networked and centralized web-accessible data archives to capture, store, and curate invasive human neurophysiological data and make them broadly available and accessible to the scientific community for furthering neuroscience research. Moreover, DABI supports investigators by providing a centralized platform to organize, compare, analyze, and share vast arrays of data collected in intracranial human recording studies in one centralized location.

There is significant need for a centralized data archive for human invasive recordings and research involving such data on various neurological and neuropsychiatric disorders. The process of analyzing such data is often multifactorial and crosses multiple modalities, and investigators require access to a large number of high quality, well-curated data points and study subjects. Since data generating and collecting sites are spread among different laboratories, clinical sites, heterogeneous data types, and formats, before the data can even be analyzed, they must be standardized, and tools for searching, viewing, annotating, and analyzing them must be coupled to those data. We have described how we have addressed these challenges while building DABI to include data de-identification, data quality assessment, analysis results data integration, mapping of data attributes into a common schema for use in search and visualization interfaces, interfaces to search, select, and download data, integrated processing so that database and compute resources are coupled, a visualization interface for inspecting and comparing data, and a comprehensive website containing training materials, study-related information (e.g., protocols), announcements, and a feedback knowledgebase wherein investigators post questions and receive answers from DABI and the community. We have also shown the power of integrated analyses with an example from representative DABI datasets.

## Data Availability

The datasets supporting the results of this article are available in the Data Archive for the BRAIN Initiative (DABI), 10.18120/sr2n-gz34^30^ (PI: Walker) and 10.18120/x7mj-am06^32^ (PI: Pouratian).

## Code availability

Open-source software was used for analysis, including the open-source H2O library for machine learning frameworks, Jupyter notebooks for python analysis, and R Analysis and Visualization of intracranial EEG (RAVE).

This project is supported by the NIH/NINDS under award number R24MH114796. Data used in the sample analyses presented were supported by the NIH/NINDS under award numbers UH3NS100553, R01NS119520 and U01NS098961 and the Michael J. Fox Foundation under grant number 15098.
